# Therapeutic Potential of Bioactive Compounds from *Brugmansia suaveolens* Bercht. & J. Presl

**DOI:** 10.3390/nu15132912

**Published:** 2023-06-27

**Authors:** Sandro Pinheiro da Costa, Raphaela Aparecida Schuenck-Rodrigues, Verônica da Silva Cardoso, Simone Sacramento Valverde, Alane Beatriz Vermelho, Eduardo Ricci-Júnior

**Affiliations:** 1 Faculty of Medicine, Centro Universitário Serra dos Órgãos, Teresópolis 25964-004, RJ, Brazil; sandropinheiropharma@gmail.com; 2 Faculdade de Farmácia, Universidade Federal do Rio de Janeiro, Rio de Janeiro 21941-902, RJ, Brazil; raphaschuenck@gmail.com; 3 Instituto de Microbiologia Paulo de Góes, Universidade Federal do Rio de Janeiro, Rio de Janeiro 21941-902, RJ, Brazil; verocardoso@micro.ufrj.br (V.d.S.C.); abvermelho@micro.ufrj.br (A.B.V.); 4 Laboratório de Química Medicinal de Produtos Bioativos, Instituto de Tecnologia em Fármacos, Rio de Janeiro 21040-900, RJ, Brazil; laqmed.fiocruz@gmail.com

**Keywords:** *Brugmansia suaveolens* Bercht. & J. Presl, Solanaceae, tropane alkaloids, therapeutic potential

## Abstract

*Brugmansia suaveolens* Bercht. & J. Presl has been widely used due to the presence of different bioactive compounds. This review summarizes the latest advances and perspectives of the *B. suaveolens* plant species; it is a systematic literature review on aspects of botany, traditional uses, phytochemistry, pharmacology, and toxicology as therapeutic potential. In addition, 120 compounds are described, including alkaloids, flavonoids, terpenoids, steroids, amino acids, aromatics, and aliphatics. As for the therapeutic potential, it is described in extracts and compounds in the antitumor, anti-inflammatory, antioxidant, antimicrobial, antispasmodic, anticoagulant, and analgesic aspects, as well as the effects on the central nervous system. The toxicity of the genus stands out, especially the potential for organ toxicity. Therefore, this review evidenced the knowledge related to the traditional use based on the scientific research of *Brugmansia suaveolens*, highlighting an overview of bioactive compounds and biological and toxicological activities in order to provide a scientific basis for future studies on the value of this species for the development of new natural products.

## 1. Introduction

Medicinal plants have been used as inexhaustible sources of new substances with potential therapeutic effects. Chemical and pharmacological studies of natural products have been the focus of many surveys in the scientific world, aiming at the discovery of new compounds with therapeutic activity due to the high costs of research and the elaboration of synthetic medications. There are several plant species capable of generating research and development based on the claim of a given therapeutic effect, which can become a valuable tool for the discovery of new drugs [1,2].

The Solanaceae family has approximately 150 genera and 300 cataloged species. This family holds species of great economic importance worldwide; most of its species are found in tropaneal areas such as Brazil. It is considered the third most economically important plant family and the first among vegetables. Some examples of these species are tomato (*Solanum lycopersicum*), with major importance in agriculture, and, for the pharmaceutical industry, *Atropa belladonna* stands out, among others [3,4].

The species from this family can be easily found in homes and gardens, such as the “trumpet tree” (*Brugmansia suaveolens* Bercht. & J. Presl), widely cultivated as an ornamental piece due to its characteristic odor and the beauty of its flowers. Within the research scope, plants belonging to the Solanaceae family are known to produce tropane alkaloids, a group of commonly toxic secondary metabolites used as plant defense [4,5].

*Brugmansia suaveolens* Bercht. & J. Presl is used in folk medicine for therapeutic purposes and in Peruvian religious myths to seek changes in the individual’s conscious state. Popularly, its dried flowers and leaves are used to treat strong coughs and bronchitis by inhaling its vapor. In the form of juice and/or ointment, it is applied to burns, abrasions, inflammation, hemorrhoids, arthritis, and rheumatism on the affected areas to relieve the pain generated by this trauma [6,7,8].

Previous studies show that species from this genus have tropaneal alkaloids such as scopolamine and atropine. The toxic action of this genus occurs due to the anticholinergic action of alkaloids, which are acetylcholine antagonists at muscarinic receptors, inhibiting the action of this transmitter in autonomic effectors and smooth muscles, decreasing mucous secretions, and blocking the action of the myocardial vagus nerve, providing increased heart rate [9,10,11].

Ingestion of this plant species in high doses makes the tropane alkaloids stimulate the central nervous system, causing depression of the peripheral nerves, which can cause some adverse effects such as disorientation, hallucinations, and panic. In more severe cases, the individual presents with neurological depression and cardiovascular and respiratory disorders, which may precede death [7,11].

Brazilian diversity contributes to the existence of a wide biodiversity, becoming an example of balanced ecosystems that provide a variety of still-explored plant communities. *B. suaveolens* Bercht. and J. Presl is still a little-reported species regarding its biological effects, which enable the elucidation of substances of natural origin, stimulating the discovery of new products with different applications. This lack of comprehensive reports enables unprecedented studies using substances of natural origin, stimulating the discovery of new products with different applications [4,8,12].

Currently, the search for new active molecules in biodiversity has intensified, and different active ingredients have been used for the development of new effective and safe technologies; however, many of these substances have an effective biological action but high toxicity. An alternative for reducing toxic substances is the development of nanopharmaceuticals in order to reduce systemic adverse effects, resulting in better adherence to the treatment. Different secondary metabolites, such as alkaloids, are oftentimes associated with different adverse effects in clinical practice due to their biological effects. Technological development represents a paradigm shift with the potential to reduce its unwanted effects by improving administration and application and minimizing toxicity [13].

This review seeks to provide an overview of the bioactive compounds from *B. suaveolens* Bercht. & J. Presl, aiming to support the evaluation of biological and toxic effects based on the presence of tropane alkaloids, a class of secondary metabolite that is promising in terms of its therapeutic application as a bioactive molecule.

## 2. Materials and Methods

This qualitative approach study reviewed the literature in question for better understanding the bioactive compounds related to the *Brugmansia suaveolens* plant species and their therapeutic applications. It was decided to carry out a systematic review, defined as an instrument for obtaining, identifying, analyzing, and summarizing the literature directed to the specific theme. It also allows for a broad literature review, including discussions of methods and publication results. Articles, monographs, dissertations, and books published on the subject matter were consulted in the SciELO, Science Direct, PubMed, and Medline databases.

To identify the study designs, the terms *Brugmansia suaveolens*, Solanaceae, and Tropane alkaloids were used. The pre-selection of the studies was based on reading the titles and/or abstracts and, when necessary, the full texts. The use of the articles was analyzed by consensus, rejecting those that did not present specific data about the research. Articles were obtained based on studies of the Solanaceae family, genus *Brugmansia*, *Brugmansia suaveolens* plant species, and the *Datura suaveolens* synonym. Of the 301 articles analyzed, 193 were excluded from the research because they did not present specific content for carrying out the work, and 108 presented essential data for carrying out the bibliographic survey.

The data will be reported following the recommendations set forth in the JBI Manual for Evidence Synthesis and PRISMA for Scoping Reviews (PRISMA ScR) and initially presented through a flowchart recommended by PRISMA ScR to indicate the evidence search flow. Subsequently, tables will be presented with information extracted from the articles included, taking into account the population, concept, and context. From the analysis of the tables, graphs will be prepared to present the correlations obtained in a didactic way. After presenting the data, they will be discussed in depth in order to list future research gaps and the limitations of the studies that will serve as a basis for further research focused on the analysis of this review.

## 3. Results and Discussion

**Figure 1:**
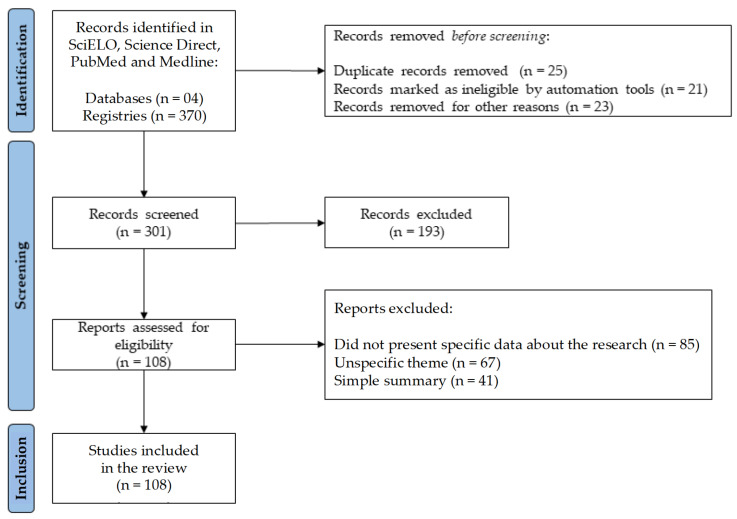
Flowchart with the study selection steps adapted from the Preferred Reporting Items for Systematic Reviews and Meta-Analyses (PRISMA).

After the selection process, 108 studies met the inclusion criteria. The study selection process is shown in a flow diagram (Figure 1), according to the PRISMA standards.

### 3.1. Medicinal Plants and Their Bioactive Compounds

Currently, pharmaceutical products are considered a promising market that moves a large part of the world economy with constant product innovations. In this context, natural products have wide applicability in the pharmaceutical market, in the production of new drugs, and in other economic sectors [14]. Nature is the biggest producer of known organic substances. Natural products offer a wide variety of bioactive molecules with great diversity in their structures and biological activities [15].

Different methods can be used to synthesize secondary metabolites with therapeutic action. Plant species can be used in these processes. Many of these substances can be highly toxic and may even be carcinogenic [13,16,17].

Secondary metabolites play an important role in the interaction between the environment and its defense against invaders. Vegetation has a wide variety of secondary metabolites, which are synthesized from primary metabolites (e.g., carbohydrates, lipids, and amino acids). These compounds are necessary in the defense against herbivores, pathogens, and environmental stresses [18]. They also have characteristics that contribute to the plants’ specific odors, tastes, and colors [19].

These substances have numerous applications, such as food additives, flavors, and industrially important products such as the development of new drugs [20]. Some of the natural products derived from plants include drugs such as morphine, codeine, cocaine, pilocarpine, and steroids such as diosgenin, digoxin, and digitoxin [8,21].

Medicinal plants are considered a great source of phytochemical compounds given their therapeutic activity, which enables the development of new drugs. Most natural compounds are of plant origin, such as phenolic substances and flavonoids, which are used in cancer treatment and prevention as well as for their antioxidant activity [22].

The interest in using natural sources in the development and formulation of skin care products such as antioxidant, photoprotective, and anti-aging products is considered an alternative to conventional cosmetic products and phytomedications, contributing to the increasing interest in research and industrial application of medicinal plants [23,24,25]. The medications derived from medicinal plants used in clinical practice in recent years are presented in Table 1.

In the context of discovering new herbal substances, an advantageous approach is essential when applied to samples from regions marked by high biodiversity and endemism, as the chemical diversity of natural products can reflect the biodiversity of their organisms of origin [49,50].

The ethnopharmacological approach is a study where the use of traditional medicine with medicinal plants constitutes the basis for the selection of test materials and pharmacological assays. Ethnopharmacology involves the observation, description, and experimental investigation of traditionally used drugs and their bioactivities. It represents a transdisciplinary concept based on botany, chemistry, biochemistry, and pharmacology [25,51,52].

### 3.2. Brugmansia Suaveolens Bercht. & J. Presl

The Solanaceae family has approximately 100 genera and 2300 cataloged species. As a holder of species of world economic importance, most of its species are found in tropaneal areas such as Brazil, where it occupies a prominent place among economically important plants. Some examples of these species are tomato (Solanum lycopersicum) and, for the pharmaceutical industry, Atropa belladonna [3].

*Brugmansia suaveolens* Bercht. & J. Presl (Humb. & Bonpl. ex Willd.), considered a botanical synonym of Datura suaveolens (Humb. & Bonpl. ex Willd.), is a plant mainly used by indigenous peoples and credited with having originated in the Andes. It grows in Peru, Bolivia, and Ecuador in the Solanaceae family, genus Brugmansia, and species suaveolens; its growth is in the form of shrubs, reaching approximately 3–9 m in height, or even more in favorable conditions. It has white or pink flowers but presents color variations; it is aromatic, in the shape of a trombette, and can measure up to 15–50 cm, from where its popular name arises, “Trompete”, as well as other popular names such as “Saia Branca”, “Cartuchiller”, “Canudo”, or “Zabumba” [3,4].

Its leaves vary from 15–30 cm in length and approximately 10 cm in width; the best type of soil for its cultivation is in humid places, it is easily found near rivers, and the time of year for its harvest and the intensity of exposure to sunlight can significantly interfere with greater or lesser yields of tropane alkaloids [3].

The Brugmansia species is native to South America. Previously, this species was considered a subgenus of Datura; however, more recent research shows that it should be classified within a genus of its own. Its popular use and wide distribution in the Americas demonstrate its relationship with men [53].

### 3.3. Tropane Alkaloids

Tropane alkaloids are named after nightshades and feature a tropane ring consisting of the pyrrolidine and piperidine rings. Tropanes have a bicyclic structure called the tropane ring. Nearly 150 tropane alkaloids are known, most of which are pyrrolidine derivatives such as hygrine and cuscohygrine, and the main ones are atropine, hyoscyamine, scopolamine, and cocaine. Atropine and scopolamine are potent anticholinergic agents used in therapy in the form of sulfate salts for ophthalmic use and as gastrointestinal relaxants [54,55,56].

Among the active pharmaceutical ingredients that have the tropane portion in their structure, the most significant in terms of volume and production value are those of natural origin, including atropine, hyoscyamine, and scopolamine. It is a group of its semi-synthetic derivatives that can be obtained by a single or more chemical steps, which can result in the formation of quaternary ammonium salts or undergo other chemical modifications or substitutions of functional groups. The biggest product in terms of production of this tropane active ingredient is scopolamine butylbromide (original preparation: Buscopan^®^, usually in soft capsules or coated tablets of 10 mg manufactured by Boehringer Ingelheim) with indications for problems of the intestinal tract, particularly as an antispasmodic agent [57,58,59].

Drugs containing tropane alkaloids are therapeutically indicated against colics in the ureters and those caused by kidney stones, bronchial spasms, cases of bronchial asthma, spasms of the gastrointestinal tract, and gastric hypersecretion. This group of substances is also used as local anesthetics, as they act by desensitizing nerve endings [56].

The biosynthesis of alkaloids takes place through metabolic pathways that have not yet been fully biochemically delineated due to the fact that many of the enzymes involved in several steps have not yet been isolated and characterized. The formation of the heterocyclic system of alkaloids normally occurs through simple inter- or intra-molecular reactions. In general, alkaloids are formed from amino acids. The alkaloids of this group are esters of acids derived from the phenylalanine amino acid by a rearrangement process [55,56].

*Brugmansia suaveolens* Bercht. & J. Presl present different secondary metabolites, which include alkaloids, steroids, phenolic compounds, terpenes, triterpenes, and flavonoids, among others, related to therapeutic potential [4,60,61]. The main metabolites are the tropane alkaloids related to significant biological activities and recognized as the main secondary metabolites in medications derived from plants [62].

**Figure 2:**
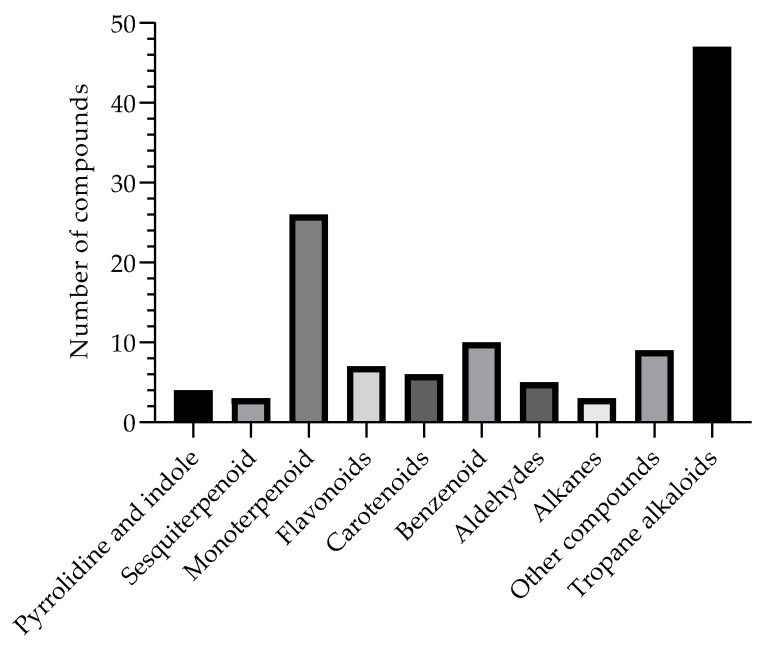
Compounds isolated and described in the *B. suaveolens* Bercht. & J. Presl plant species obtained, compiled as source data for the review.

These alkaloids represent 40% of all compounds isolated from the genus *B. suaveolens* Bercht. & J. Presl, therefore also helping as chemotaxonomic markers; in this way, the compounds found in this species and in their respective organs are presented in Table 2, as they have a wide spectrum of therapeutic activities. Among the reports in the literature, Figure 2 represents the compounds described and elucidated in the literature on the species in question, including 47 tropane alkaloids; 04 pyrrolidine and indole; 03 sesquiterpenoids; 26 monoterpenoids; 07 flavonoids; 06 carotenoids; 10 benzenoid compounds; 05 aldehydes; 03 alkanes; and another 09 elucidated compounds, mainly found in calculus, roots, leaves, flowers, and seeds, demonstrating countless possibilities of new biologically active compounds, thus being considered a promising alternative for the chemistry of natural products.

**Figure 3:**
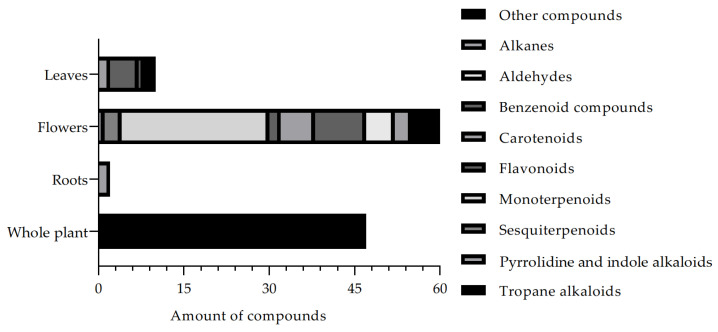
Compounds isolated and described in the *B. suaveolens* Bercht. & J. Presl plant species obtained, compiled as source data for the review.

Based on the description of the compounds isolated from the *B. suaveolens* Bercht. & J. Presl plant species, Figure 3 shows the list of the main phytochemical constituents described in the literature. Thus, this review allows us to observe that Tropane alkaloids are the most frequently described compounds in the literature, followed by Monoterpenoids, respectively, corroborating data from the traditional use of the plant species.

**Figure 4:**
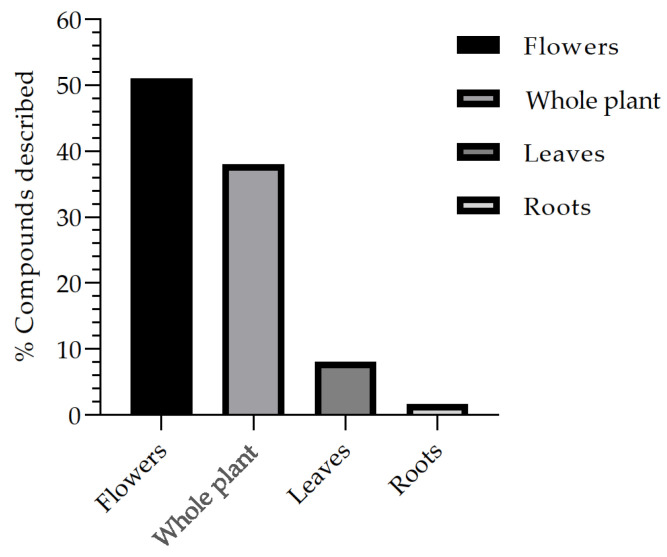
Frequency of the main parts of the *B. suaveolens* Bercht. & J. Presl plant species used for compound isolation studies and biological assays.

Another fact that can be observed through the review is the frequency of compounds isolated and described through the bibliographic survey, in which the percentage (%) is presented based on the total number of studies analyzed in this review (Figure 4). Thus, it is possible to describe that the compounds commonly isolated from the different organs of the plant and described in studies based on extraction, isolation, and biological studies are the flowers, which represent 51% of the studies, followed by the whole plant with 38% and the leaves with 8%, while the roots represent 1.6%, being respectively associated with the *B. suaveolens* Bercht. & J. Presl plant species. These data allow for contributing to a broad spectrum of research on flowers, such as risks in a variety of compounds, whereas for roots, further research should be encouraged to expand knowledge and related secondary metabolites.

### 3.4. Therapeutic Applications of Tropane Alkaloids from Brugmansia spp.

Plants from the genus *Brugmansia,* as well as other Solanaceae, have alkaloids with wide therapeutic applications. Scopolamine and its derivatives have parasympatholytic, anticholinergic, antiemetic, and sedative actions; these substances are mainly used as pre-anesthetics. Due to their action, they become mydriatic and cycloplegic agents, with a mechanism similar to atropine [1,4,12].

*Brugmansia* species differ in the concentration of atropine and scopolamine, both CNS depressants with sedative and tranquilizing properties, relevant ophthalmic action, and in the salivary, bronchial, and sweat glands. One of the adverse effects of scopolamine is drowsiness, which can also produce excitement and hallucinations. These effects are similar to those caused by toxic doses [4,12], although scopolamine can still be used as a heroin detoxifying agent without causing dependence [12].

Regarding pharmacodynamics, scopolamine differs only quantitatively from atropine. While atropine has almost no detectable CNS effects at clinically applicable doses, scopolamine exerts prominent CNS effects at low therapeutic doses. This difference can be explained by better penetration of scopolamine into the blood–brain barrier [4,12].

The medicinal properties of plants can be based on phytochemical effects such as antioxidant, antimicrobial, and antipyretic activity. In this way, medicinal plants can be considered potent and promising therapeutic agents for the improvement of processes such as wound healing based on their variety of active and effective components, including flavonoids, alkaloids, phenolic compounds, and terpenoids. These metabolites can be adhered to as modern therapy due to their low cost, limited adverse effects, bioavailability, and efficacy. The emergence and development of nanoscience and technology can help improve the effectiveness of different therapies. Thus, nanoformulations have advantages over conventional therapy, providing a unique opportunity to ease the treatment of skin lesions, even for chronic wounds, and providing an efficient and fast healing process, resulting in reduced hospitalization costs [81].

Traditional uses of *Brugmansia* and bioactive compounds have led to improved validation of the species’ therapeutic potential. These extracts have been shown to have a wide range of pharmacological properties. Table 3 shows the traditional uses and biological activities of the *B*. *suaveolens* Bercht. & J. Presl species.

#### 3.4.1. Anti-Inflammatory Activity

Although many traditional use reports mention that the genus *Brugmansia* has different pharmacological properties, there is little research directing the proven biological activities to support these traditional uses. Recently, the anti-inflammatory activities of extracts from *B. suaveolens* flowers and leaves were evaluated through cellular electrophoretic analysis of NF-kB, p38α, TNFα and elastase. The data showed that the ethanol extract (100 μg/mL) from *B. suaveolens* inhibited NF-kB binding to DNA. The extract also shows inhibition of the p38α action with a value of 54.86 ± 2.82 μg/mL. These extracts directly altered elastase activity, with a value of 51.35 ± 0.69 μg/mL. In the elastase assay by human neutrophils, they showed release (65.98 ± 1.84 μg/mL) [106]. Although this study presents several in vitro tests to investigate the anti-inflammatory activity of different parts of *B*. *suaveolens*, further pharmacological and phytochemical studies are required to precisely identify which isolated compound is responsible for the anti-inflammatory effect.

#### 3.4.2. Cytotoxic Activity

The evaluation of the cytotoxicity of extracts from *B. suaveolens* leaves and flowers was analyzed by colometry assay through reaction with (3-(4,5-dimethylthiazol-2-yl)-2,5-diphenyltetrazolium bromide (MTT). The results suggest that extracts (100 μg/mL) from the plants show cytotoxic effects with a value of 92 ± 0.7 μg/mL [106]. However, the constituents of the extracts must be elucidated by bioguided isolation to define the cytotoxic relationship. Furthermore, phytochemical studies of the ethanolic extract from *B. suaveolens* leaves led to the isolation of a new monoterpenoid with immunomodulator-mediated antitumor activity. The results showed that this monoterpenoid increased the secretion of IFN-γ, an immunological marker, and of IL-2 from peripheral blood mononuclear cells. They therefore observed an increase in the number of cell deaths in the A549 and MCF7 cell lines and showed an increase in ROS production and mitochondrial membrane disruption leading to apoptosis [8,80].

#### 3.4.3. Antispasmodic Activity

The antispasmodic activity of stem and leaf extracts from *B. suaveolens* was evaluated on smooth muscle contraction in rabbits. The results showed that the extract (71.5 μg/mL) had a significant antispasmodic effect [107]. Together, the presence of tropane alkaloids in this species may explain the traditional use of these plants as antispasmodics. However, more research is necessary to isolate the bioactive compound with antispasmodic activity from these herbal extracts.

#### 3.4.4. Antibacterial Activity

The antibacterial activity of methanolic extracts from *B. suaveolens* stem, leaves, and flowers was evaluated by means of the disk diffusion test. The result showed that extracts from the stem of the plant exerted mild antibacterial activity, while extracts from the leaves and flowers had no antibacterial activity [8,108].

#### 3.4.5. Anti-Asthmatic Activity

Anisa et al. [109] performed an in vivo investigation of the antiasthmatic activity of the aqueous extract from the *B*. *suaveolens* leaves. Vogel’s method was used to record breathing patterns, and the aqueous extract was administered orally after being dissolved in distilled water. The results showed that the dose of 40 mg/kg of body weight of the aqueous extract exerted a significant effect when compared to salbutamol sulfate at a dose of 0.16 mg/kg of body weight. The presence of tropane alkaloids in the plant extract may explain its antiasthmatic activity and corroborate its traditional use.

#### 3.4.6. Antinociceptive Effects

The antinociceptive effects of the *B. suaveolens* flower extract were investigated using hotplates, muscle contractions, a formalin assay, and tail movement experiments in murines. The extracts were dissolved and administered intraperitoneally. The extract doses (100 and 300 mg/Kg) showed an increase in latency by the hotplate method, inhibiting the abdominal constrictions induced by acetic acid and producing the hypnotic effect generated by pentobarbital in a dose-dependent manner. Furthermore, both doses inhibited the formalin test phase. Similarly, the Tail-flick test shows attenuation of the response. The results suggest that the extract from *B*. *suaveolens* flowers has antinociceptive activity related to popular reports of the plant species [110,111,112]. However, further research studies are required, seeking bioguided isolation in the identification of the bioactive compounds related to the antinociceptive activity.

#### 3.4.7. Antiprotozoal Activity

The in vitro antileishmanial activity of extracts from *B. candida* and *B. suaveolens* species was tested against *Leishmania amazonensis* promastigotes. The findings showed that extracts from *B. suaveolens* flowers (86.2 ± 9.5 μg/mL) and leaves (33.9 ± 2.3 μg/mL) exerted antileishmanial activity, while *B. candida* extracts showed no antiprotozoal activity [113]. The results are in line with the traditional use of the species for the treatment of antiprotozoa, skin infections, and ulcers. However, complementary assays are necessary for better data robustness, thus seeking bioguided monitoring of the bioactive compounds present in the extracts.

### 3.5. Toxicity

It is known that species from the genus *Brugmansia* are commonly associated with toxicity due to the presence of different alkaloids, among which the presence of atropine and scopolamine in different organs stands out [75,114]. The concentrations of these alkaloids vary according to seasonality, nutritional status, and organ; thus, in the leaves, there are concentrations of atropine (0.79 ± 0.03 mg/g) and scopolamine (0.72 ± 0.05 mg/g) in dry presentation, while the scopolamine concentrations in nectar are increased in the flowers (149.80 ± 6.01 μg/mL) [75,115]. The main symptoms most related to toxicity are dry and red dermis, pupil dilation, hallucinations, headaches, hysteria, dry mouth, tachycardia, arrhythmias, fever, epilepsy, urinary incontinence, and other anticholinergic symptoms [116].

The *B. suaveolens* species represented one of the main ornamental plants responsible for poisoning in humans from 1992 to 2009, with the highest percentage rate (5.71%) among toxic species [117,118]. The systemic effects of poisoning by this species are similar to those caused by the atropine alkaloid from the belladonna species, which has high hallucinogenic power and causes several health problems with the possibility of leading to death [119].

According to Antony et al. (2009), the *B. suaveolens* flowers are also made up of essential oil, presenting a different tonality according to time alteration: before fully opening, they are yellow; at dusk, they are already fully open and white; and the next day, they are pink. The study carried out with the white flowers revealed the presence of several essential oil constituents, with 1,8-cineole (72.1%), €-nerolidol (11.7%), and α-terpineol (5.3%) identified as the main compounds. The pink flowers showed megastigmatrienone II (24.5%), nonanal (17.4%), terpinen-4-ol (10.5%), and a series of long linear hydrocarbons. These hydrocarbons were also identified in the white flowers in low concentrations, but megastigmatrienone II was not identified.

Consequently, there are numerous reports of accidental poisoning by species from the genus *Brugmansia* [109]. Therefore, further studies using in vivo models are required to evaluate the toxicological profile in order to determine the minimum effective dose of the extract and clarify the changes and elucidation of pharmacodynamic and pharmacokinetic models associated with newly identified and isolated compounds from this species.

## 4. Conclusions

This review is a study focused on the traditional uses, secondary metabolites, biological activity, and toxicity of *Brugmansia suaveolens* Bercht. & J. Presl, capable of dazzling the promising therapeutic potential of this species and making it possible to establish scientific grounds to subsidize future studies on the species. Thus, this paper contributes to understanding current knowledge and gaps in the bioactive compounds found in *Brugmansia suaveolens*. It also points the way for the design of comprehensive studies to further explore the composition of active and relevant phytochemicals in this species.

Therefore, this review evidenced knowledge related to the traditional use based on fundamental scientific research about *Brugmansia suaveolens* Bercht. & J. Presl, highlighting an overview of bioactive compounds and biological and toxicological activities in order to provide the scientific grounds for future studies on the value of this species for the development of new therapeutic agents.

## Figures and Tables

**Table 1:** Medications derived from medicinal plants in clinical practice in recent years.

Compound Name/Commercial Name	Chemical Structure	Chemical Class	Species	Botanical Family	Indication	Mechanism	Year	Refs
Artemisinin	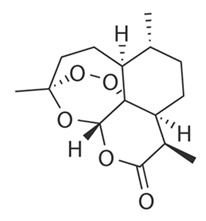	Sesquiterpene lactone	*Artemisia tenuisecta* *Artemisia annua* L.	Asteraceae	Malaria treatment	Radical formation	1987	[26,27]
Arglabin^®^	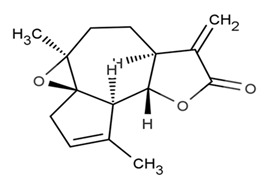	Sesquiterpene	*Artemisia myriantha* *Artemisia obtusiloba* var. *glabra* Leeb	Asteraceae	Cancer, colon, ovarian and lung cancer	Farnesyl transferase inhibition	1999	[28,29]
Capsaicin Qutenza^®^	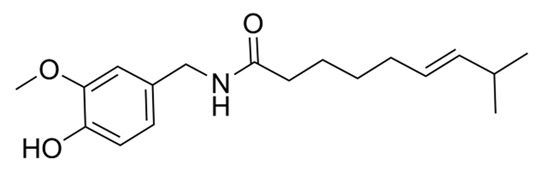	Capsaicinoid	*Capsicum annum* L., *C.mill mínimo* *Capsicum pubescens*	Solanaceae	Neuropathic pain (topical analgesic)	TRPV1 agonist. Na-channel blocker.	2010	[30,31]
Colchicine Colcrys^®^	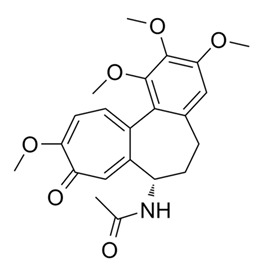	Alkaloid	*Colchicum* spp.	Colchicaceae	Calcific tendinitis, gout, arthritis	Tubulin binding. CYT P450 3A4 inhibitor p-glycoprotein interaction.	2009	[32,33]
Dronabinol Cannabidol Dronabinol Sativex^®^	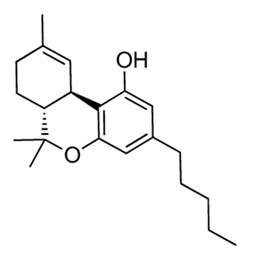 Dronabinol (delta9-THC) 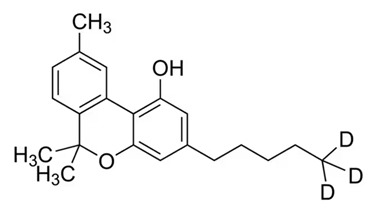 Cannabidol (CBD)	Cannabinoids (diterpenoid)	*Cannabis sativa* L.	Cannabaceae	Chronic neuropathic pain, chemotherapy-associated nausea and anorexia nervosa cachexia	Activation of CB1 and CB2 receivers	2005	[34,35]
Galantamine Razadyne^®^	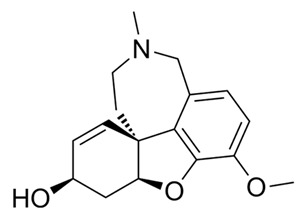	Alkaloid	*Galanthus caucasicus* (Baker) Grossh. *Galanthus nivalis*	Amaryllidaceae	Dementia associated with Alzheimer’s disease, mild to moderate	Acetylcholinesterase inhibitor. Ligand of human nicotinic acetylcholine receptors (nAChRs)	2001	[36,37]
Ingenol Mebutate Picato^®^	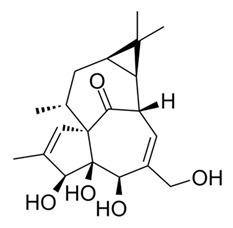	Alkaloid	*Euphorbia peplus* L.	Euphorbiaceae	Actinic keratosis	Cell death inducer	2012	[38,39]
Masoprocol Actinex^®^	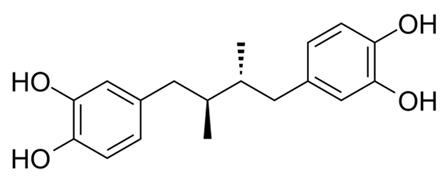	Terpene	*Larrea tridentata* (Sessé & Moc. Ex DC.) Coville	Zygophyllaceae	Cancer chemotherapy	Lipoxygenase inhibitor	1992	[40,41]
Omacetaxine Mepesuccinate Synribo^®^	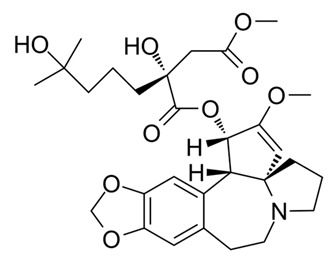	Alkaloid	*Cephalotaxus harringtonia*	Cephalotaxaceae	Oncology	Protein transcription inhibitor	2012	[42,43]
Paclitaxel Taxol^®^ Abraxane^®^ Nanoxel^®^	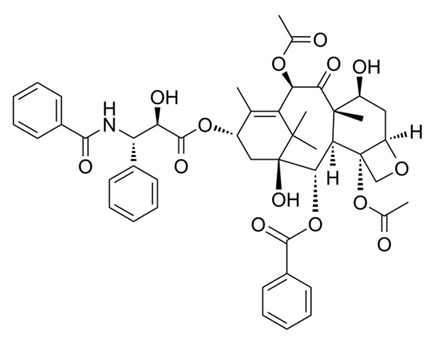	Diterpene	*Taxus brevifolia* Nutt	Taxaceae	Cancer chemotherapy	Mitotic inhibitor	1971	[44,45]
Solamargine Curaderm^®^	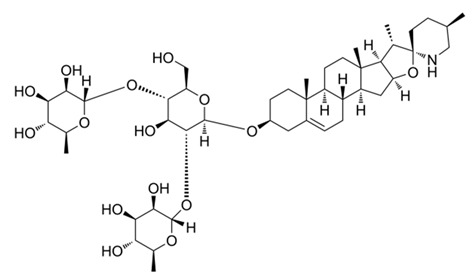	Flavonoid	*Solanum* spp.	Solanaceae	Cancer chemotherapy	Triggering Apoptosis	1989	[46,47,48]

**Table 2:** Compounds found in *Brugmansia suaveolens* Bercht. & J. Presl.

No.	Compounds	Formula	Part	References
Tropane alkaloids				
01	3-(3′-Acetoxytropoyloxy)-tropane	C_19_H_25_NO_4_	Whole plant (root, stem, leaf, fruit, flowers and seeds)	[63]
02	Apoatropine	C_17_H_21_NO_2_		[63,64]
03	Atropine	C_17_H_23_NO_3_		[5,9,10,11,65,66,67]
04	Hyoscyamine	C_17_H_23_NO_3_		[11,63,67,68]
05	Littorine	C_17_H_23_NO_3_		[6,63,67]
06	Noratropine	C_16_H_21_NO_3_		[67,69]
07	Norhyoscyamine	C_16_H_21_NO_3_		[69]
08	3α-Phenylacetoxytropane	C_16_H_21_NO_2_		[63]
09	3-(Hydroxyacetoxy)-tropane	C_10_H_17_NO_3_		
10	6-Hydroxyacetoxytropane	C_10_H_17_NO_3_		
11	3β-Tigloyloxytropane	C_13_H_21_NO_2_		
12	3-Tigloyloxynortropane	C_12_H_19_NO_2_		
13	3α-Acetoxytropane	C_10_H_17_NO_2_		[67,70]
14	Pseudotropine	C_8_H_15_NO		[63,71]
15	Tropine	C_8_H_15_NO		[6,63]
16	3α-tropanol	C_8_H_15_NO		[69,72]
17	3α-Apotropoyloxy-6β-hydroxytropane	C_17_H_21_NO_3_		[63]
18	3,6-Dihydroxytropane	C_8_H_15_NO_2_		[63,69]
19	3α,6β-Ditigloyloxytropane	C_18_H_27_NO_4_		
20	3β,6β-Ditigloyloxytropane	C_18_H_27_NO4		[63]
21	3α-Hydroxy-6β-acetoxytropane	C_10_H_17_NO_3_		
22	3-Hydroxy-6-(2-methylbutyryloxy)-tropane	C_13_H_23_NO_3_		
23	3α-Hydroxy-6β-tigloyloxytropane	C_13_H_21_NO_3_		[63,69]
24	6-Hydroxyhyoscyamine	C_17_H_23_NO_4_		[63]
25	7-Hydroxyhyoscyamine	C_17_H_23_NO_4_		
26	3-Hydroxy-6-methylbutyryloxytropane	C_13_H_23_NO_3_		
27	3-Isovaleryloxy-6-hydroxytropane	C_13_H_23_NO_3_		[63,69]
28	3-Phenylacetoxy-6-hydroxytropane	C_16_H_21_NO_3_		[63]
29	3α-Tigloyloxy-6β-hydroxytropane	C_13_H_21_NO_3_		[63,69]
30	3-Tigloyloxy-6-propionyloxytropane	C_16_H_25_NO_4_		[63]
31	3α-Tigloyloxy-6β-isobutyryloxytropane	C_17_H_27_NO_4_		
32	3-Tigloyloxy-6-(2′-methylbutyryloxy)-tropane	C_18_H_29_NO_4_		
33	3,7-Dihydroxy-6-tigloyloxytropane	C_12_H_19_NO_4_		[63,69]
34	3α,6β-Ditigloyloxy-7β-hydroxytropane	C_18_H_27_NO_5_		[63,67]
35	3-Tigloyloxy-6-propionyloxy-7-hydroxytropane	C_16_H_25_NO_5_		[63]
36	3α-Tigloyloxy-6β-isovaleryloxy-7β-hydroxytropane	C_18_H_29_NO_5_		[63,67]
37	3β-Tigloyloxy-6β-isovaleryloxy-7β-hydroxytropane	C_18_H_29_NO_5_		[63]
38	Meteloidine	C_13_H_21_NO_4_		[63,67]
39	Aposcopolamine	C_17_H_19_NO_3_		[6,63,67]
40	Apohyoscine	C_17_H_19_NO_3_		[67,69]
41	Hyoscine	C_17_H_21_NO_4_		[67,68,69,73]
42	Norhyoscine	C_16_H_19_NO_4_		[65,67,69]
43	Norscopolamine	C_16_H_19_NO_4_		[67,69]
44	3-Phenylacetoxy-6,7-epoxynortropane	C_15_H_17_NO_3_		[63]
45	Scopolamine	C_17_H_21_NO_7_		[63,65,67,74,75,76]
46	Scopoline	C_8_H_13_NO_2_		[6,63,77]
47	Scopine	C_8_H_13_NO_2_		[63,77]
Pyrrolidine and indole alkaloids				
01	Cuscohygrine	C_13_H_24_N_2_O	Roots	[67,77]
02	Indole	C_8_H_7_N	Roots Flowers	[70,71]
03	3-(3-indolyl) lactic acid	C_11_H_11_NO_3_	Leaves	[78]
04	3-(3-indolyl) lactic acid methyl ester	C_13_H_15_NO_2_		
Sesquiterpenoids				
01	*trans,trans-Farnesol*	C_15_H_26_O	Flowers	[70,71]
02	Farnesal	C_15_H_24_O		[67]
03	(*E*)-Nerolidol	C_15_H_26_O		[7]
Monoterpenoids				
01	Allo-ocimene	C_10_H_16_	Flowers	[70]
02	Citronellal	C_10_H_18_O		[70,79]
03	Citronellol	C_10_H_20_O		
04	Geranial	C_10_H_16_O		
05	Geraniol	C_10_H_18_O		
06	Geranyl acetate	C_12_H_20_O_2_		[79]
07	Linalool	C_10_H_18_O		[7,79]
08	β-Myrcene	C_10_H_16_		[70,79]
09	Neral	C_10_H_16_O		
10	*Cis*-β-Ocimene	C_10_H_16_		
11	(*E*)-β-Ocimene	C_10_H_16_		[7]
12	*Cis*-Ocimenol	C_10_H_18_O		[79]
13	*trans*-Ocimenol	C_10_H_16_O		
14	*trans*-β-Ocimene	C_10_H_16_		[70,79]
15	α-Pinene	C 10H16		[70]
16	β-Pinene	C_10_H_16_		
17	α-Thujene	C_10_H_16_		
18	Sabinene	C_10_H_16_		
19	*trans-Sabinene* hydrate	C_10_H_18_O		
20	1,8-Cineole	C_10_H_18_O		
21	Limonene	C_10_H_16_		
22	α-Terpineol	C_10_H_18_O		
23	Terpinolene	C_10_H_16_		
24	Terpinen-4-ol	C_10_H_18_O		[7]
25	γ-Terpinene	C_10_H_16_		
26	SUPH036-022A	C_13_H_14_O_5_		[80]
Flavonoids				
01	Kaempferol	C_15_H_10_O_6_	Flowers	[9]
02	Kaempferol 3-O-β-D-glucopyranosyl-(1‴ →2″)-O-α-L-arabinopyranoside	C_26_H_28_O_15_		[12]
03	Kaempferol 3-O-β-D-glucopyranosyl-(1‴ →2″)-O-α-L-arabinopyranoside-7-O-β-D-glucopyranoside	C_32_H_38_O_20_	Leaves	
04	Kaempferol 3-*O*-β-D-[6‴-*O*-(*E*-caffeoyl)]-glucopyranosyl-(1‴ →2″)-*O*-α-Larabinopyranoside-7-*O*-β-D-glucopyranoside	C_41_H_44_O_23_		
05	Kaempferol 3-O-β-D-[2‴-O-(Ecaffeoyl)]-glucopyranosyl-(1‴ →2″)-O-α-L-arabinopyranoside-7-O-β-D-glucopyranoside	C_41_H_44_O_23_		
06	Kaempferol 3-O-L-arabinopyranoside	C_20_H_18_O_10_		
07	Kaempferol 3-O-L-arabinopyranosyl-7-O-D-glucopyranoside	C_26_H_28_O_15_		
Carotenoids				
01	Megastigmatrienone I	C_13_H_18_O	Flowers	[7]
02	Megastigmatrienone II	C_13_H_18_O		
03	Megastigmatrienone III	C_13_H_18_O		
04	Megastigmatrienone IV	C_13_H_18_O		
05	Theaspirane A	C_13_H_22_O		
06	Theaspirane B	C_13_H_22_O		
Benzenoid compounds				
01	Benzyl alcohol	C_7_H_8_O	Flowers	[70,79]
02	Benzaldehyde	C_7_H_6_O		
03	Benzyl benzoate	C_14_H_12_O_2_		
04	Benzyl salicylate	C_14_H_12_O_3_		
05	4-Methoxy benzaldehyde	C_8_H_8_O_2_		
06	Methyl benzoate	C_8_H_8_O_2_		
07	Methyl salicylate	C_8_H_8_O_3_		
08	Phenylacetaldehyde	C_8_H_8_O		
09	Phenylethyl alcohol	C_8_H_10_O		[7,71]
10	3-phenyllactic acid	C_9_H_10_O	Leaves	[78]
Aldehydes				
01	Decanal	C_10_H_20_O	Flowers	[70]
02	Hexanal	C_6_H_12_O		
03	Heptanal	C_7_H_14_O		[7]
04	Nonanal	C_9_H_18_O		
05	Octanal	C_8_H_16_O		
Alkanes				
01	Hentriacontane	C_31_H_64_	Flowers	[7]
02	Nonacosane	C_29_H_60_		
03	Pentacosane	C_25_H_52_		
Other compounds				
01	Physalindicanol A	C_28_H_46_O_2_	Leaves	[78]
02	Physalindicanol B	C_28_H_46_O_2_		
03	20-hydroxyecdysone	C_27_H_44_O_7_	Flowers	[9]
04	Acanthoside B	C_28_H_36_O_13_		
05	Scopoletin-7-O-β-D-galactopyranoside	C_16_H_18_O_9_		
06	2-Isobutyl-3-methoxypyrazine	C_9_H_14_N_2_O		[7]
07	6-Methyl hept-5-en-2-one	C_8_H_14_O		[67,79]
08	Hexanol	C_6_H_14_O		[70]
09	(Z)-3-Hexen-1-ol	C_6_H_12_O		

**Table 3:** Therapeutic potential of *Brugmansia suaveolens* Bercht. & J. Presl and its bioactive compounds.

Popular Traditional Use						
Popular Name	Region	Part	Form of Preparation	Traditional Use		References
Pink wandug, Maikua	Ecuador	Leaves, stem and root	-	Hallucinogenic		[82,83,84]
Toe, Misha colambo	Peru	Leaves	Maceration, decoction, juice and ointment	Gastric disorders, hallucinogenic, menstrual cramps, infections, wounds, ulcers, body pain, rheumatic pain and vaginal antiseptic		[85,86,87]
Trombeteira Canudo	Brazil	Flowers	Infusion	Gastric disorders, hallucinogenic, infections, wounds, ulcers, body pain, rheumatic pain		[60,88]
Floripon	Argentina and Mexico	Leaves and flowers	Hot oil	Boils, dermatological diseases		[89]
-	Dominica	Flowers	Cigarette	Hallucinogenic		[90]
Fleur trompete	Mauritius	Flowers	Cigarette and inhalation	Anti-asthmatic and bronchial problem		[91]
-	Philippines	Flowers	Infusion and ointment	Cough, anti-asthmatic and wounds		[92]
Padaing Kucubung	Indonesia	Leaves, flowers and seeds	-	Sedative and anti-asthmatic, gonorrhea, inflammation, intoxication and loss of appetite		[90,93,94,95,96]
Gangmeto	Bhutan	Leaves	Infusion and ointment	Hallucinogenic		[97]
Shaitani	Pakistan	Leaves	-	Stomach pain, ulcer, ringworm, body pain, rheumatic pain, skin infection and diarrhea		[98]
Bakha tobowo, Dhatura	India	Leaves, flowers and seeds	Decoction, ointment, inhalation and decoction	Wounds, rheumatic pain, body pain, swelling, cough, asthma, nasal congestion, sinusitis		[99,100,101,102,103,104,105]
Screening of pharmacological activity						
Activity	Parts	Preparation	Concentration	Model and assay	Effect	Reference
Anti-inflammatory	Leaves and flowers	Ethanolic and n-hexanoic extracts	100 μg/mL	In vitro studies show changes in electrophoretic mobility for NF-kB, p38α, TNF-α and elastase assays	Extracts inhibit NF-kB DNA binding, p38α activity, and directly impair elastase activity	[106]
Cytotoxicity	Leaves and flowers	Ethanolic and n-hexanoic extracts	100 μg/mL	Colorimetric assay (in vitro)	Extracts showed cytotoxic activities	
		Patent number 130SUPH036-022A	-	Analysis of cell viability and reactive oxygen species. Study of the cell cycle and levels of IFN-γ and IL-2	Antitumor activity by immunomodulation	[80]
Antispasmodic	Stem and leaves	Ethanolic extract	71.5 μg/mL	In vitro analysis and smooth muscle contraction	Antispasmodic activities enhanced by the action of the extract.	[107]
Antibacterial	Stem, leaves and flowers	Methanolic extract	5 μL	In vitro analysis by disk diffusion technique	Stem extracts show antibacterial activity	[108]
Anti-asthmatic	Leaves	Aqueous extract	40 mg/kg body weight	In vivo, examining the anti-asthmatic action of the extract in the guinea pig	The extract showed considerable anti-asthmatic activity	[109]
Antinociceptive	Flowers	Aqueous extract	100 and 300 mg/kg body weight	In vivo, hotplate, abdominal-writhing, sleep, formalin, and tail-flick experiments in mice	The extract presents significant antinociceptive potency	[110,111,112]
Antileishmanial	Flowers, leaves and stem	Hydroalcoholic extract	200 μg/mL	In vitro test on *L. amazonensis* promastigotes	The extract from flowers and leaves presents antileishmanial activity	[113]

## Data Availability

Not applicable.
